# Assessment of organochlorine pesticide residues in agricultural soils of southern Nigeria and analysis of potential health risks

**DOI:** 10.1016/j.toxrep.2024.101843

**Published:** 2024-11-30

**Authors:** Imeobong U. Udoekpo, Akwaowo I. Inyangudoh, Treasure A. Awa-Arua, Ekeoma I. Ogwo, Nnanake-Abasi O. Offiong, Edu J. Inam, Crispin J. Halsall

**Affiliations:** a Department of Chemistry, University of Uyo, Uyo, Nigeria; b Centre for Energy and Environmental Sustainability Research (CEESR), University of Uyo, Uyo, Nigeria; c Department of Chemistry, Faculty of Physical Sciences, Akwa Ibom State University, Ikot Akpaden, Akwa Ibom State, Nigeria; d Lancaster Environment Centre, Lancaster University, Lancaster LA1 4YQ, UK; e Department of Chemical Sciences, Faculty of Computing & Applied Sciences, Topfaith University, Mkpatak 530109, Nigeria

**Keywords:** Agricultural soils, Organochlorine pesticide, Public health, Risk assessment, Southern Nigeria

## Abstract

The use of pesticides in commercial farms can lead to exposure among various vulnerable groups. This study assessed the distribution, human health risks, and origins of 13 targeted organochlorine pesticides (OCPs) in soil samples from commercial farms in Southern Nigeria. Following a questionnaire survey on pesticide usage, soil samples were subjected to Soxhlet extraction and analyzed using gas chromatography-mass spectrometry. Results indicated that 31.6 % of farmers did not use personal protective equipment, and only 37.6 % had received training on safe pesticide application. While pyrethroids and organophosphates were commonly used, organochlorines were rarely applied. The detected levels of OCPs in the agricultural zones ranged from below detection limit (BDL) to 18.35 ± 13.83 µg/kg and were generally within the minimum risk level (MRL), except for α-HCH and β-HCH in Oron and Etinan zones. The estimated non-carcinogenic risk s from detected OCPs were insignificant at the time of the study, as the calculated Hazard Indexes (HIs) and Hazard Quotients (HQs) for ingestion, dermal absorption, and inhalation were all less than 1, indicating generally low risks. The carcinogenic risk assessment showed that the Incremental Lifetime Cancer Risk (ILCR) values ranged from 10^−10^ to 10^−4^, indicating a very low to low risk level according to the classification by the United States Agency for Toxic Substances and Disease Registry (US ATSDR). Source apportionment suggested that most OCP congeners were of historical origin, with only a few indicating recent use. In conclusion, organochlorine pesticide residues in the studied farms posed minimal health risks, with most originating from historical rather than recent use.

## Graphical Abstract

**Figure:**
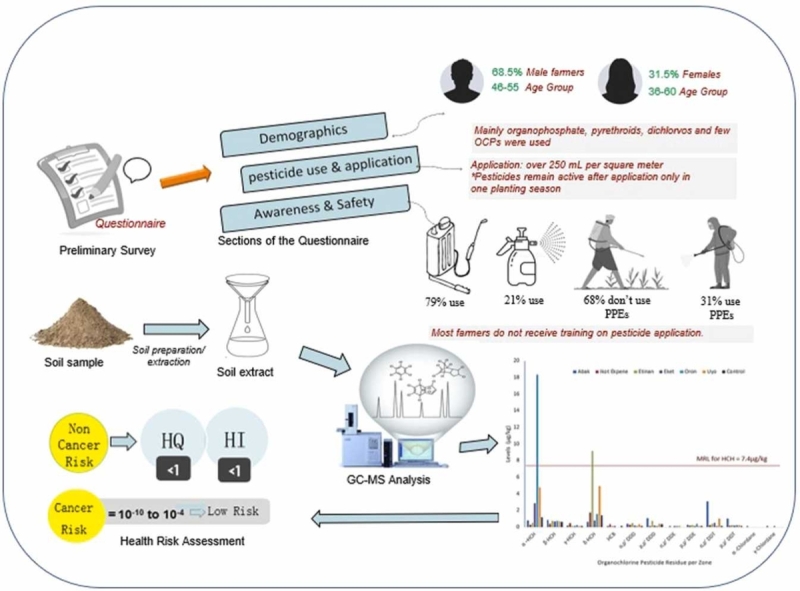

## 1 Introduction

Pest control remains one of the most effective strategies for enhancing crop yields. However, the adverse effects of certain pesticide classes on agricultural ecosystems have raised significant concerns in recent years 1, 2. Until the 1980s, organochlorine pesticides (OCPs) were the primary choice for insect pest control. Their usage declined due to international restrictions and increasing insect resistance, leading to a shift toward other pesticide classes such as organophosphates and carbamates 3, 4. According to data from the Food and Agricultural Organization (FAO), pesticide use, particularly herbicides, has risen substantially in many West African countries since the early 2000s 3, 5, 6. Despite restrictions, OCPs are still use in controlling malarial mosquitoes, with their illicit application in agriculture still prevalent across West Africa [7]. OCPs are a class of agrochemicals widely utilized in agriculture, households, and public health initiatives 8, 9. OCPs are considered as persistent organic pollutants (POPs) by the Stockholm Convention and their use are restricted in many parts of the world due to public health and environmental concerns 10, 11, 12. These chemicals are characterized by high toxicity, volatility, persistence, and bioaccumulation 13, 14, 15. They are hydrophobic, prevalent, and resistant to degradation with high potential to accumulate in soil, plant and fatty tissues of organisms 16, 17, 18. In agricultural development, pesticides became an important chemical tool for plant protection and for enhancing crop yield. According to [18], pest infestations reduce annual food production by 45 %, highlighting the need for effective pest control to combat pests and increase agricultural output. The robust economic growth of the late 19th century drove a surge in the production and use of agrochemicals, often with negative environmental consequences 18, 19. Despite existing government regulations, these pesticides remain in use in many developing and underdeveloped countries, including Nigeria 20, 21. Consequently, OCP contamination has become a significant national and global concern due to its toxicity and long-term risks to human health and the environment. Exposure to chemical pollutants can occur through various pathways such as inhalation, dermal contact, and ingestion 22, 23. Pesticide exposure may increase the incidence of type 2 diabetes and associated co-morbidities, especially for organochlorines and its metabolites [24]. Studies have also reported that individuals with Parkinson's disease and a family history of the condition are more likely to develop the disease at a younger age when exposed to metals and pesticides over extended periods [25]. The link between pesticides exposure and cancer has been widely documented 26, 27, 28, 29, 30, 31, 32. Certain OCPs have been independently linked to breast cancer due to their oestrogenic effects on mammary cells [33]. Additionally, a study measuring plasma concentrations in men and women found that individuals with high levels of OCPs in their plasma were approximately three times more likely to experience cognitive impairment in the future compared to those with low plasma concentrations [34].

As in many other developing nations, resource-poor rural farmers in Nigeria rely heavily on chemical pesticides 35, 36. In most low-income countries, especially in Africa 37, 38, safe practices for handling, storing, and applying pesticides are not widely adopted 39, 40, 41, 42, and Nigerian farmers are no exception. A review of environmental distribution of organochlorine pesticides in Africa reveals that Nigeria is the most affected country in the continent 11, 43, 44, suggesting a potentially high rate of usage of restricted pesticides. A further survey of literature reveals that previous studies in southern Nigeria are focused on secondary polluted environments such as sediments and surface waters 11, 20, 45, 46, ignoring the primary source which is the farmland soils where they are often applied and the possibility of soil to plant transfers and human uptake. Quantifying OCP residues in agricultural soils which is a major contamination source [47], is essential to mitigate health risks, raise awareness, promote sustainable agriculture, and protect ecosystems, supporting key Sustainable Development Goals (SDGs) like 3, 14, and 15. The aim of this study was to: (i) examine pesticide application practices and safety among local farmers; (ii) quantify the levels and distribution of 13 organochlorine pesticides (OCPs); (iii) assess human health risks; and (iv) determine the potential sources of OCPs in soil samples from commercial farms in southern Nigeria, providing insights into exposure risks and the historical and recent contributions to OCP contaminatio**n.**

## 2 Materials and methods

### 2.1 Description of the study area

**Fig. 1:**
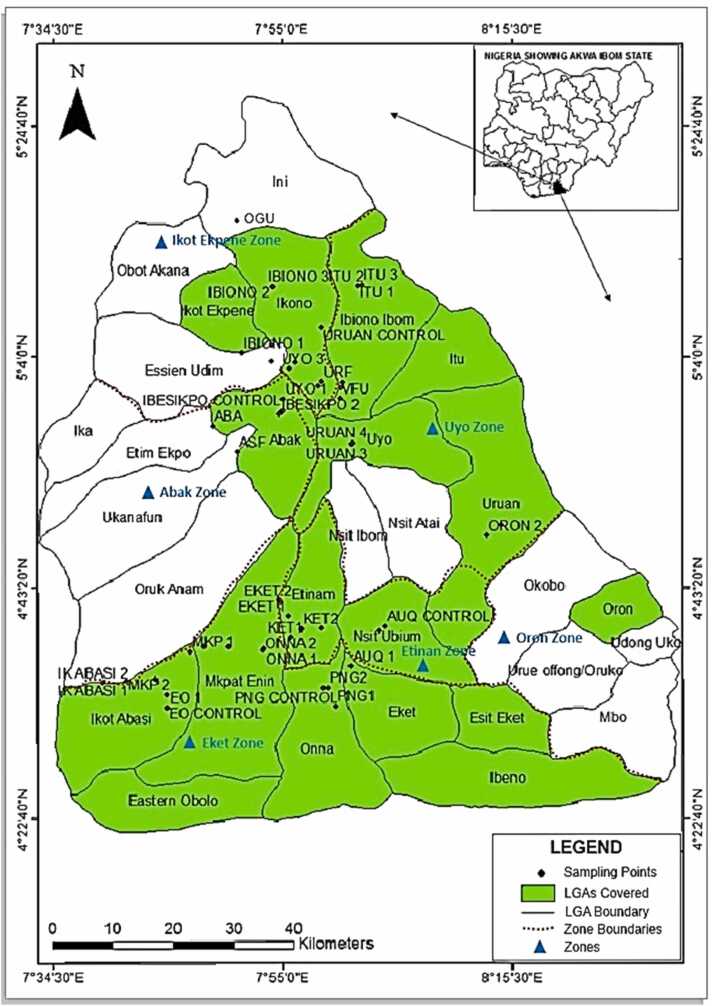
Akwa Ibom state of Nigeria showing the studied agricultural zones and locations of monitored commercial farms.

The study was conducted in six agricultural zones of Akwa Ibom State, a major cassava-producing region in southern Nigeria (Fig. 1). Akwa Ibom State is located between 4°32′N and 5°33′N, and 7°25′E and 8°25′E, in the coastal southern part of the country, covering a total area of 7081 km². As one of Nigeria's thirty-six states, Akwa Ibom is home to over 5 million people and is divided into six agricultural zones. These zones were established to facilitate the administration of government-sponsored rural agricultural projects, such as those supported by the World Bank 48, 49. The study area was chosen due to increased agricultural activities following recent government policies on agricultural development in the state.

### 2.2 Survey of pesticide use in the study area

The study employed questionnaire interviews with farmers to gain insights into current pesticide use practices and identify commonly used products. The interviews were conducted in the Local Government Areas of Uyo, Abak, Ikot Ekpene, Etinan, Eket, and Oron. A total of 486 farmers were randomly selected, with their willingness to participate being essential for the success of the survey. Pre-survey discussions with leaders of the Farmer's Cooperative Union in each community helped facilitate the process.

A structured questionnaire was developed to collect data on pesticide usage, risk perceptions, attitudes towards labels, safety precautions, farmers' knowledge, and signs of illness related to pesticide exposure. Data were gathered through face-to-face interviews, conducted in both English and local languages (Ibibio, Annang, Ekid, and Oro), which are commonly spoken by the farmers. The following headings guided the design of the interview questionnaire:

1) General information: This section collected data on farmers' biodata, including sex, age, household size, education level, and farm location and size.
2) Pesticide use and practices: This section gathered information on the types of pesticides used, farmers' attitudes toward pesticide labels, pesticide applicators and suppliers, and the use of protective equipment.
3) Human and environmental impacts: This section focused on the effects of pesticide exposure, including common illness symptoms during spraying operations, as well as trends in biodiversity changes, such as alterations in weed, bird, and insect populations (whether increasing, decreasing, or stable).

### 2.3 Soil sampling and pre-treatment

Sample collection and preparation followed the method proposed by Ogbeide et al. [50]. Sampling was conducted on five commercial farmlands in each agricultural zone. At each site, five soil grab samples (0–5 cm depth) were randomly collected using a stainless-steel hand trowel, prewashed with acetone, and then combined to form a composite sample. This gives a total of 30 composited samples comprising 150 grab samples. In addition, five grab samples were collected from farmlands where farmers reported no pesticide application; these samples were treated as controls. The collected soil samples were placed in double-layered aluminum foil, wrapped, and stored in labeled Ziploc bags. The composite soil samples were then transported to the laboratory and stored at 4°C before pre-treatment and analysis.

Sample extraction and quantification were done targeting thirteen OCPs: including α-hexachlorocyclohexane (α-HCH), β-hexachlorocyclohexane (β-HCH), γ-hexachlorocyclohexane (γ-HCH), δ-hexachlorocyclohexane (δ-HCH), Hexachlorobenzene (HCB), o,p’ and p,p’ dichlorodiphenyldichloroethane (DDD), o,p′ and p,p′ dichlorodiphenyldichloroethylene (DDE), o,p′ and p,p′ dichlorodiphenyltrichloroethane (DDT), as well as α-chlordane and γ-chlordane. Exactly 300 mL of dichloromethane (DCM) was measured into a clean round-bottom flask, and anti-bumping granules were added. A Soxhlet extractor was set up, switched on, and allowed to run for 16 hours. Each sample was rotary evaporated, transferred to a 7.5 mL vial with three washings using n-hexane, and further reduced under nitrogen (N_2_) to 1 mL.

### 2.4 Instrumental analysis

The extracts obtained were analyzed using Gas chromatography/Mass spectrometry (Trace GC Ultra - DSQ) (Xcalibur software Version 1.4.x) operating in electron impact mode (70 eV) and equipped with an Agilent CP-Sil 8 CB 50 m × 0.25 mm capillary column with 0.12 μm film thickness. The quantification was done using a 10-point mixed calibration standard in n-dodecane (10 −450 pg/μL for OCPs, 10 −120 pg/μL for PCBs, and 10 −1250 pg/μL for PBDEs, respectively).

The equipment was operated at 250 °C in both the single ion monitoring (SIM) and electron impact (EI) modes, with chemical concentrations reported in this study corrected for recovery but not blank corrected. The oven temperature was initially set at 70 °C and held there for two minutes. It was then raised to 150 °C at a rate of 25 °C/min, raised again to 220 °C (3 °C/min), and lastly raised to 300 °C (10 °C/min) and held there for ten minutes. A quality control (QC) standard which involves a calibration range of 2–200 pg/g was used to guarantee instrument precision and recoveries ranged from 70 % to 110 %.

### 2.5 Quality control and quality assurance

For each set of samples, the method detection limit and procedural blank were assessed. The presence of analytes in the method blanks enabled the identification of target compounds in the samples, based on retention times matching those of reference standards within specified limits, ranging from 2 to 10 ng/kg. The method detection limits (MDLs) are defined as the mean concentration of the blank plus three times the standard deviation. The concentration of OCPs were reported in µg/kg with values less than 0.01 µg/kg were recorded as below-detection-limits (BDL) and zero during data treatment. Similarly, the analysis of samples was done in triplicates and the results are presented as mean values and standard deviation.

### 2.6 Risk assessment procedure

#### 2.6.1 Non-carcinogenic risk

(1), (2), (3), (4), (5), (6) were used to calculate the average daily dosage (ADD) (mg/kg/day), hazard quotient (HQ), and hazard index (HI) of the pollutant through various exposure pathways to assess non-carcinogenic risk [51]. In these equations, C_soil_ represents the concentration of OCP residue in the soil sample, and RfD is the reference dose. The risk assessment method is based on standard guidelines from the United States Environmental Protection Agency (USEPA) for both adults and children, which were adopted for this study. Additionally, the risk estimation focuses solely on agricultural soil as the route of exposure, excluding food consumption and drinking water.

(1) $$\mathrm{ADD}\mathrm{ingest}=\frac{\begin{array}{c} C_{\mathrm{soil}}\;\times \mathrm{IngR}\times \mathrm{EF}\times \mathrm{ED}\times \mathrm{CF} \end{array}}{\mathrm{BW}\times \mathrm{AT}}$$

(2) $$\mathrm{ADD}\mathrm{dermal}=\frac{\begin{array}{c} C_{\mathrm{soil}}\;\times \mathrm{SA}\times {\mathrm{AF}}_{\mathrm{soil}}\;\times \mathrm{ABS}\times \mathrm{EF}\times \mathrm{ED}\times \mathrm{CF} \end{array}}{\mathrm{BW}\times \mathrm{AT}}$$

(3) $$\mathrm{ADD}\mathrm{inhale}=\frac{\begin{array}{c} C_{\mathrm{soil}}\;\times \mathrm{InhR}\times \mathrm{EF}\times \mathrm{ED} \end{array}}{\mathrm{PEF}\times \mathrm{BW}\times \mathrm{AT}}$$

(4) $$\mathrm{HQ}=\frac{\begin{array}{c} \mathrm{ADD} \end{array}}{\mathrm{RfD}}$$

(5) HI=ƩHQi

(6) Total exposure hazard index(HI)=ƩHQi

Where ADD_ingest,_ ADD_dermal_ and ADD_inhale_ represent the Average Daily Dose for oral ingestion, dermal absorption, and inhalation, respectively. The specific values of other variables are detailed in Table S1 (supplementary Information) 17, 52.

The reference dose (RfD) (mg/kg/day) represents the maximum tolerable daily dose, with children considered a particularly sensitive group. It serves as a threshold: a daily dose exceeding the RfD may lead to adverse effects over a lifetime of exposure. The reference doses and cancer slope factors for various OCPs [53] are provided in Table S2 (Supplementary Information). If the average daily dose (ADD) is lower than the RfD, the risk of adverse health effects is considered tolerable. However, if the ADD exceeds the RfD, the risk of harm increases. The hazard quotient (HQ), calculated as the ratio of ADD to RfD, indicates potential health risks: an HQ ≤ 1 suggests no adverse effects, while an HQ > 1 indicates possible harm [54].

#### 2.6.2 Cancer Risk

The incremental lifetime cancer risk (ILCR) represents the probability of an individual developing cancer due to lifetime exposure to a carcinogenic agent. The ILCR was calculated using Eq. 7. (8), (9), (10) were used to compute the ILCR for oral, dermal, and inhalation exposure pathways, following the standard models of the U.S. Environmental Protection Agency 51, 55. A description of each parameter, along with their units, is provided in Tables S1 and S2.

(7) ILCR=ADD×CSF

(8) $$\mathrm{ILCR}\mathrm{ingest}=\frac{\begin{array}{c} C_{\mathrm{soil}}\;\times (\mathrm{CS}F_{\mathrm{ingest}}\;\times \sqrt[{3}]{\left(\mathrm{BW}/70\right)})\times {\mathrm{IR}}_{\mathrm{soil}}\;\times \mathrm{EF}\times \mathrm{ED} \end{array}}{\mathrm{BW}\times \mathrm{AT}\times \mathrm{CF}}$$

(9) $$\mathrm{ILCR}\mathrm{dermal}=\frac{\begin{array}{c} C_{\mathrm{soil}}\;\times (\mathrm{CS}F_{\mathrm{dermal}}\;\times \sqrt[{3}]{\left(\mathrm{BW}/70\right)})\times \mathrm{SA}\times \mathrm{FE}\times \mathrm{AF}\times \mathrm{ABS}\times \mathrm{EF}\times \mathrm{ED} \end{array}}{\mathrm{BW}\times \mathrm{AT}\times \mathrm{CF}}$$

(10) $$\mathrm{ILCR}\mathrm{inhale}=\frac{\begin{array}{c} C_{\mathrm{soil}}\;\times (\mathrm{CS}F_{\mathrm{in}h\mathrm{ale}}\;\times \sqrt[{3}]{\left(\mathrm{BW}/70\right)})\times {\mathrm{IR}}_{\mathrm{air}}\;\times \mathrm{EF}\times \mathrm{ED} \end{array}}{\mathrm{BW}\times \mathrm{AT}\times \mathrm{PEF}}$$

where, C_soil_ is the concentration of OCP in soil (µg/kg), ADD is the Average Daily Dose and CSF is the cancer slope factor. The predicted risks were ranked using the following ILCR classification: values < 10^−6^ (very low risk); 10^−6^ < value ≤ 10^−4^ (low risk);10^−4^ < value ≤ 10^−3^ (moderate risk); 10^−3^ < value ≤ 10^−1^ (high risk); and value ≥ 10^−1^ (very high risk).

### 2.7 Source identification and apportionment

The ratios of the concentrations of parent compounds to their degradation products were calculated and used to assess the potential historical origin of OCPs in the study locations 21, 56.

## 3 Results and discussion

### 3.1 Survey of pesticide use in commercial farms in the study location

The majority (68.5 %) of the farmers interviewed were male, with ages ranging from 46 to 55 years (Table S3). Among the 486 farmers surveyed, 15 % had no formal education, and only a small percentage could read and write, likely limiting their ability to understand pesticide container labels. Most farmers relied on explanations from other farmers or pesticide providers, as they were either illiterate or had only completed primary school. Only 5.2 % of farmers reported consistently reading pesticide labels, while the remainder never read them before or after purchasing pesticides. Of the 486 respondents, 37.6 % had received formal or informal training on pesticide use and application, while 62 % had not received any training (Table S4). A wide variety of pesticide types were used in each zone, including pyrethroids, organophosphates, and carbamates, with only a few farmers reporting the use of OCPs (Table S5). The choice of pesticides depended on the information or education received, pesticide availability in the zone, and the type of crops cultivated. Pumpkin, maize, and cassava were the most important crops, listed in decreasing order of significance (Table S6).

When applying diluted formulations and handling concentrated pesticides, it is essential for applicators to wear proper personal protective equipment (PPE). Most respondents (68.4 %) reported using PPE when handling pesticides (Table S7). However, the remaining 31.6 % cited reasons for not using PPE, including lack of information, inconvenience, unavailability, and economic factors. Farmers who did not wear protective equipment typically wore clothing made of wool, cotton, or synthetic materials, which are insufficient to protect them from chemical exposure during spraying. These materials can absorb pesticide solutions, bringing chemicals closer to the applicators and increasing their risk of exposure. Additionally, it was observed that farmers used only two methods of application: 20.9 % used hand pumps, while 79.1 % used knapsack sprayers (Table S8).

Farmers believe that pesticides are effective for only one planting season after application (Table S9). Most farmers apply more than 250 mL of pesticides per square meter of farmland (Table S10). Typically, they purchase small quantities of pesticides from nearby stores convenient to their residences. As shown in Table S11, pesticides were applied once, twice, three times, or more during a planting season. Most respondents (40.6 %) applied pesticides twice per season, while 21.2 % applied them as needed. Excessive pesticide application not only degrades farmland but also negatively impacts human health and the environment. According to Table S12, 63 % of farmers send their produce to market within 2–3 weeks after pesticide application, while 24.9 % wait up to four weeks or more. However, some farmers still send their products to market just one week after application.

### 3.2 Levels of Organochlorine Pesticide Residues in soil

**Fig. 2:**
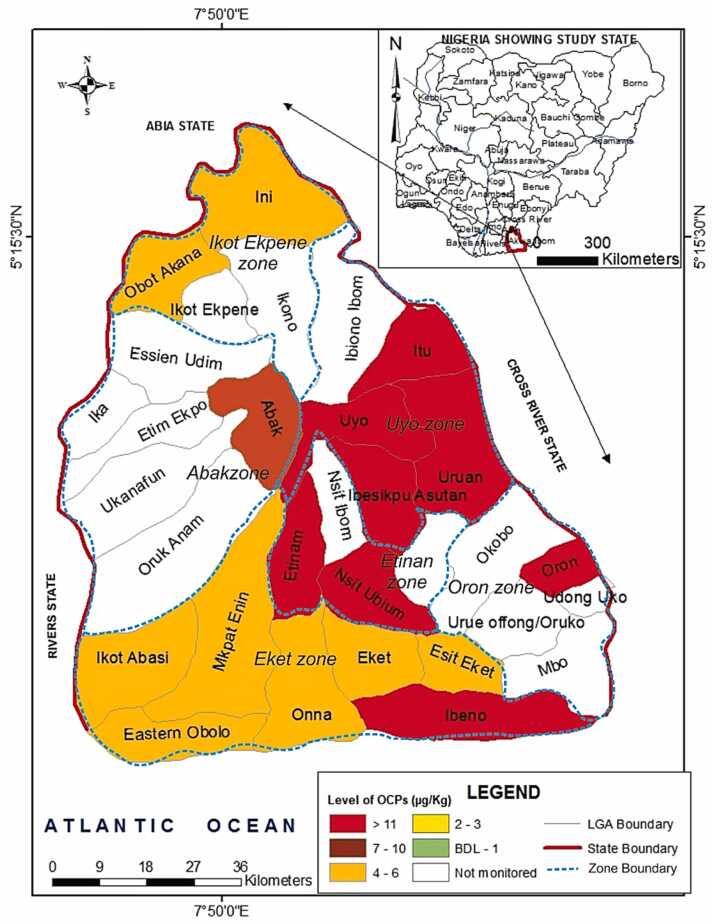
Spatial distribution of pesticide in six agricultural zones of Akwa Ibom State, Nigeria.

The levels of organochlorine pesticides (OCPs) in agricultural soils are presented in Table 1 and Fig. 2. The results showed varying concentrations of OCPs across the monitored agricultural zones. Generally, concentrations ranged from below detection limits (BDLs) for HCB, o,p’-DDE, α-chlordane, and γ-chlordane to 18.35 ± 13.83 µg/kg for α-HCH in the Oron agricultural zone. All targeted OCPs were below the US Agency for Toxic Substances and Disease Registry's minimum risk levels (MRL) [57], except for α-HCH in Oron and β-HCH in Etinan, where mean concentrations ( ± SD) were 18.35 ± 13.83 µg/kg, exceeding the 7.4 µg/kg MRL. The cumulative levels of the 13 OCPs showed 100 % detection across all monitored agricultural zones (Table 1). However, some OCPs, particularly α-chlordane and γ-chlordane, were below detectable levels in four of the six agricultural zones. Spatial distribution of OCPs revealed significantly higher total OCP levels (> 11 µg/kg) in the more populated state capital, Uyo, compared to other zones like Oron, Eket, and Ibeno (Fig. 2). The higher OCP levels in Oron and Ibeno LGAs can be attributed to their role as major entry points for imported goods and the year-round farming activities in riverine farmlands.

**Table 1:** Levels (µg/kg) of OCPs in soils of commercial farms in six agricultural zones of the study area.

OCPs	Agricultural Zones							MRL
	Abak	Ikot Ekpene	Etinan	Eket	Oron	Uyo	Control	
α –HCH	0.78 ± 0.03^a^	0.31 ± 0.12^a^	0.51 ± 0.31^a^	2.88 ± 0.89^a^	18.35 ± 13.83^b^	4.75 ± 2.06^a^	1.19 ± 0.62^a^	7.4
β-HCH	0.87 ± 0.08^a^	0.40 ± 0.40^a^	0.72 ± 0.68^a^	0.69 ± 0.20^a^	0.71 ± 0.11^a^	0.68 ± 0.35^a^	0.60 ± 0.38^a^	6.0
γ-HCH	0.19 ± 0.03^a^	0.47 ± 0.31^b^	0.12 ± 0.00^a^	0.08 ± 0.04^a^	0.22 ± 0.03^a^	0.13 ± 0.07^a^	0.11 ± 0.06^a^	9.0
δ-HCH	0.60 ± 0.05^a^	1.77 ± 0.07^a^	9.14 ± 7.42^a^	0.81 ± 0.35^a^	1.57 ± 0.37^a^	4.96 ± 2.70^a^	1.40 ± 0.46^a^	400
HCB	0.02 ± 0.00^a^	0.33 ± 0.13^b^	0.09 ± 0.09^a^	0.04 ± 0.03^a^	BDL	BDL	0.01 ± 0.00^a^	100
o,p’ DDD	0.38 ± 0.05^a^	0.27 ± 0.05^a^	0.47 ± 0.39^a^	0.19 ± 0.10^a^	0.04 ± 0.01^a^	0.32 ± 0.13^a^	0.06 ± 0.03^a^	NA
p,p’ DDD	1.07 ± 0.07^b^	0.08 ± 0.06^a^	0.71 ± 0.70^b^	0.18 ± 0.11^a^	0.03 ± 0.01^a^	0.37 ± 0.25^a^	0.33 ± 0.20^a^	3000
o,p’ DDE	BDL	0.01 ± 0.01^a^	BDL	0.09 ± 0.07^a^	0.01 ± 0.00^a^	0.03 ± 0.01^a^	BDL	NA
p,p’ DDE	0.28 ± 0.02^b^	0.18 ± 0.03^a^	0.24 ± 0.12^b^	0.09 ± 0.04^a^	0.38 ± 0.06^b^	0.08 ± 0.01^a^	0.05 ± 0.02^a^	2000
o,p’ DDT	3.11 ± 0.12^b^	0.25 ± 0.20^a^	0.37 ± 0.16^a^	0.50 ± 0.30^a^	0.12 ± 0.09^a^	1.04 ± 0.69^a^	0.02 ± 0.02^a^	NA
p,p’ DDT	1.00 ± 0.00^b^	0.18 ± 0.11^a^	0.22 ± 0.14^a^	0.20 ± 0.09^a^	0.22 ± 0.11^a^	0.24 ± 0.19^a^	0.10 ± 0.04^a^	2000
α -Chlordane	0.03 ± 0.03^b^	BDL	BDL	BDL	BDL	BDL	BDL	0.6
γ-Chlordane	0.02 ± 0.00^a^	BDL	BDL	0.04 ± 0.03^a^	BDL	BDL	BDL	0.6
∑OCPs	8.35	4.18	12.61	5.79	21.66	12.61	3.88	

MRL = minimum risk level [44]

NA = not available.

*Similar superscripts* indicate *no significant difference (p > 0.05), while different superscripts indicate a significant difference (p < 0.05)*

The environmental quality criteria used in a recent study classify the levels of HCHs and DDTs in soils as follows: ≤ 50 µg/kg (negligible contamination), 50 – 500 µg/kg (low pollution), 500 – 1000 µg/kg (moderate pollution), and > 1000 µg/kg (high pollution) [8]. Based on this classification, the contamination level of OCPs in the study area is considered "negligible." Additionally, a comparison of OCP levels from this study with reports from other countries shows that the recorded levels fall within the lower ranges of global data (Table S13). Several factors, including the history of pesticide application, agricultural practices such as irrigation, superphosphate use, and tillage, as well as soil properties like pH, total organic carbon (TOC), water content, and texture, influence the residual levels and environmental behavior of OCPs in soils. Consequently, the release of OCPs from contaminated soils remains a source of environmental pollution due to their persistence and long history of use.

The results show that o,p′-DDT, p,p′-DDT, and α-Chlordane levels in Abak were significantly higher than in other zones (p < 0.05). However, no significant differences were observed in β-HCH, δ-HCH, o,p′-DDD, o,p′-DDE, and γ-Chlordane across the zones (p > 0.05). γ-HCH and HCH levels were significantly higher in Ikot Ekpene compared to other zones (p < 0.05), while p,p′-DDD was not significantly different between Abak and Etinan (p > 0.05), but was significantly higher than in other zones (p < 0.05). Correlation analysis revealed a significant positive relationship between β-HCH and both o,p′-DDD (r = 0.440, p < 0.05) and p,p′-DDD (r = 0.588, p < 0.01). Additionally, o,p′-DDD showed a significant positive relationship with p,p′-DDD (r = 0.745, p < 0.01) and o,p′-DDT (r = 0.551, p < 0.01). p,p′-DDD was positively correlated with o,p′-DDT (r = 0.648, p < 0.01) and p,p′-DDT (r = 0.534, p < 0.01). A significant positive relationship was also found between o,p′-DDE and γ-Chlordane, while o,p′-DDT showed a significant positive correlation with both p,p′-DDT (r = 0.409, p < 0.05) and α-Chlordane (r = 0.433, p < 0.05). Furthermore, a significant positive relationship was established between α-Chlordane and p,p′-DDT (r = 0.502, p < 0.05) (Table S14).

### 3.3 Estimated risks of OCPs

The results of the risk assessment are presented in Table 2 and in Tables S15–S32 of the Supplementary file. Table 2 summarizes the hazard indexes (HIs) for all the agricultural zones studied. These HIs represent the overall and cumulative non-carcinogenic risk (∑HQs) of the targeted OCPs in the study area. The results show that the estimated non-carcinogenic risks indicate no significant adverse health effects from the detected OCPs at the time of the study, as the calculated HIs were all less than 1 (Table 2). Non-carcinogenic risks from ingestion, dermal absorption, and inhalation were generally low, with hazard quotients (HQs) and hazard indexes (HIs) below 1. However, prolonged exposure could eventually pose a risk. In contrast, the carcinogenic risk assessment revealed that the incremental lifetime cancer risk (ILCR) ranged from 10^−10^ to 10^−4^ indicating a very low to low risk level, according to the ATSDR classification [58]. The highest risk level was calculated for HCH exposure through ingestion in Oron, for both children and adults (Tables S27-S32).

**Table 2:** Estimated hazard indexes for exposures to OCPs.

Zone	ADULT_ingest_	CHILDREN_ingest_	ADULT_dermal_	CHILDREN_dermal_	ADULT_inhale_	CHILDREN_inhale_
Abak	3.29E−13	3.20E−13	1.71E−13	1.16E−13	2.76E−17	1.34E−17
Ikot Ekpene	2.35E−13	2.28E−13	1.22E−13	8.30E−14	1.97E−17	9.56E−18
Etinan	7.76E−13	7.54E−13	4.02E−13	2.74E−13	6.50E−17	3.16E−17
Eket	3.42E−13	3.32E−13	1.77E−13	1.21E−13	2.86E−17	1.39E−17
Oron	1.45E−12	1.41E−12	7.52E−13	5.13E−13	1.22E−16	5.91E−17
Uyo	7.78E−13	7.56E−13	4.03E−13	2.75E−13	6.52E−17	3.17E−17
Control	2.42E−13	2.35E−13	1.25E−13	8.56E−14	2.03E−17	9.86E−18

### 3.4 Source evaluation

Based on the isomeric ratios of OCPs (Table 3), α-HCH/γ-HCH ratios were > 3 in most agricultural zones except Ikot Ekpene. An α-HCH/γ-HCH ratio > 3 suggests the source is likely technical-HCH, possibly indicating long-range transport where α-HCH is the dominant isomer 21, 59. In contrast, the α-HCH/γ-HCH ratio < 3 in Ikot Ekpene indicates fresh or recent use of Lindane, a pesticide formulation dominated by the γ-isomer [21]. The β-HCH/γ-HCH ratio > 1 suggests historical usage, while a ratio < 1 indicates recent input. In this study, historical usage was observed in the Eket, Oron, and Uyo agricultural zones, whereas recent inputs were noted in Abak, Ikot Ekpene, and Etinan. For o,p′-DDT/p,p′-DDT, a ratio > 1 suggests dicofol as the major DDT source [21]. Among the zones, only Oron recorded an o,p′-DDT/p,p′-DDT ratio < 1. The DDD/DDE ratio was > 1 in most zones except Oron, indicating that DDD is the dominant degradation product of DDT, while in Oron, DDE is the primary degradation product. The p,p′-DDT/ƩDDT ratio ranged from 0.19 to 0.65 across the six agricultural zones (Table 3). A ratio > 0.5 signifies aged DDT use. Notably, only the Oron agricultural zone recorded a p,p′-DDT/ƩDDT ratio > 0.5, indicating long-term DDT application in the soils.

**Table 3:** Isomeric ratios of Pesticide Residues Across the Zones.

OCPs	Abak	Ikot Ekpene	Etinan	Eket	Oron	Uyo
*α-*HCH/*γ-*HCH	4.11	0.66	4.25	36.00	83.41	36.54
*α-*HCH /*β-*HCH	0.90	0.78	0.71	4.17	25.85	6.99
*o,p′* DDT/*p p′* DDT	3.11	1.39	1.68	2.50	0.55	4.33
DDD/DDE	5.18	1.84	4.92	2.06	0.18	6.27
*p,p′* DDT/ƩDDT	0.24	0.42	0.37	0.29	0.65	0.19

This study provides a comprehensive survey of pesticide use among commercial farmers in southern Nigeria with a case study of Akwa Ibom State, focusing on pesticide types, usage patterns, farmer knowledge, and personal protective equipment practices. It also provides insights into the levels of organochlorine pesticide residues in soil, offering risk assessments with low carcinogenic non-carcinogenic and risks. However, the study has limitations, including lack of long-term monitoring and lack of data on effectiveness of local regulation. Despite the large sample size, there is a possibility that the study on pesticide types and usage frequency might not fully capture the extent of pesticide misuse or the use of banned chemicals, especially in regions where farmers may be unwilling to disclose illegal practices. Furthermore, the findings are limited to Akwa Ibom State, and while the study provides valuable insights into pesticide use and contamination in this region, the results may not be directly applicable to other regions with different agricultural practices, pesticide usage habits, and environmental conditions. Further studies in different regions would help generalize the findings.

## 4 Conclusion

This study found that commercial farmers in the study area use various pesticide classes, including organochlorines (OCPs), organophosphates, pyrethroids, and dichlorvos, some of which are banned or restricted in the EU and other regions. While OCP residues were generally low in the sampled soils, an exception was α-HCH in Oron, which measured 18.35 ± 13.83 µg/Kg—exceeding the US ATSDR minimum risk level of 7.4 µg/Kg. Despite this, the risk assessment for human health through soil exposure showed negligible non-carcinogenic risk, as hazard quotients (HQs) and hazard indices (HIs) were below 1 for all pathways. The estimated carcinogenic risk was also low across the agricultural zones. Diagnostic ratios and survey responses suggested that OCPs in soils likely stem from historical use rather than recent applications. However, most farmers had no formal training on pesticide use, and many cited economic constraints, inconvenience, lack of information, and unavailable protective equipment as reasons for not using safety gear, pointing to potential exposure risks and environmental contamination routes.

Key findings include:

1. Farmers use various pesticides, some of which are banned or restricted.
2. Non-carcinogenic health risks from pesticide exposure were negligible.
3. Carcinogenic risk levels across the zones were very low to low.
4. OCP contamination likely originates from past applications, not current widespread use.
5. Most farmers lack training and face barriers to adopting protective measures.

Further research should monitor long-term pesticide residue levels, assess ecosystem impacts, and evaluate the outcomes of farmer training on pesticide safety practices.

## Funding

The Tertiary Education Trust Fund (TETFUND) Institutional Based Research grant 2012–2015 combined intervention provided funding for this study.

## CRediT authorship contribution statement

**Imeobong U. Udoekpo:** Writing – review & editing, Writing – original draft, Resources, Methodology, Formal analysis, Data curation. **Akwaowo I. Inyangudoh:** Writing – review & editing, Writing – original draft, Resources, Investigation, Data curation. **Treasure A. Awa-Arua:** Writing – review & editing, Writing – original draft, Validation, Resources, Investigation, Formal analysis, Data curation. **Ekeoma I. Ogwo:** Software, Resources, Methodology, Investigation, Formal analysis, Data curation, Conceptualization. **Nnanake-Abasi O. Offiong:** Writing – review & editing, Validation, Project administration, Methodology, Formal analysis, Data curation, Conceptualization. **Edu J. Inam:** Writing – review & editing, Supervision, Project administration, Methodology, Funding acquisition, Conceptualization. **Crispin J. Halsall:** Writing – review & editing, Supervision, Resources, Project administration, Methodology, Funding acquisition.

## Declaration of Competing Interest

The authors declare that this study was not influenced by any known financial interests or personal relationships that could present a competing interest.

## Data Availability

Data will be made available on request.
