# Supplementary material for: Assessment of organochlorine pesticide residues in agricultural soils of southern Nigeria and analysis of potential health risks
Source: Toxicol Rep. 2024 Nov 30;13:101843. doi: 10.1016/j.toxrep.2024.101843 (PMC11665699; doi:10.1016/j.toxrep.2024.101843)
Supplement: Table S1 — Supplementary material [file mmc1.docx]

**Assessment of organochlorine pesticide residues in agricultural soils of southern Nigeria and analysis of potential health risks**

**Supplementary Information**

**Table S1: Summary of risk assessment variables**

| **Exposure parameters** | **Unit** | **Child** | **Adult** |
| --- | --- | --- | --- |
| Body weight (BW) | Kg | 13.95 | 58.75 |
| C_soil_ (level of OCP in soil) | mg/kg |  |  |
| Ingestion rate (IngR or IR_soil_) | mg/d | 200 | 100 |
| Exposure frequency (EF) | d/yr | 350 | 350 |
| Exposure duration (ED) | Yr | 6 | 30 |
| Average life span (AT) | D | LT × 365 | LT × 365 |
| Lifetime (LT) | Yr | 72 | 72 |
| Surface area (SA) | cm^2^/d | 2800 | 5700 |
| Dermal exposure ratio (FE) | Unitless | 0.61 | 0.61 |
| Dermal surface factor (AF) | mg/cm | 0.2 | 0.07 |
| Dermal absorption factor (ABS) | Unitless | 0.13 | 0.13 |
| Inhalation rate (InhR_air_) | m^3^/d | 10.9 | 17.5 |
| Particle emission factor (PEF) | m^3^/kg | 1.36 × 10^9^ | 1.36 × 10^9^ |

Source: Qu et al [17, 52]

Table S2: Reference Dose (RfD) and Cancer Slope Factor (CSF) for OCPs

| OCPs | RfD (ingest, dermal, inhale) | CSF_ingest_ | CSF_dermal_ | CSF_inhale_ |
| --- | --- | --- | --- | --- |
| HCH | 2.00E-4 | 6.3 | 4.49 | 6.3 |
| DDD | 5.00E-4 | 0.24 | 0.343 | NA |
| DDE | 5.00E-4 | 0.34 | 0.486 | NA |
| DDT | 5.00E-4 | 0.34 | 0.486 | 0.34 |
| HCB | 8.00E-4 | 1.6 | 3.2 | NA |
| Chlordane | 5.00E-4 | 0.35 | 0.7 | 0.35 |

Source: Man et al [53]

Table S3: Demographics of the survey

|  | Uyo  (n=24) | IkotEkpene  (n=63) | Abak  (n=51) | Etinan  (n=35) | Eket  (n-43) | Oron  (n=53) | Total  (n=486) |
| --- | --- | --- | --- | --- | --- | --- | --- |
| *Gender* |  |  |  |  |  |  |  |
| Male | 173(71.8) | 39(61.9) | 37(72.5) | 18(51.4) | 27(62.8) | 39(73.6) | 333(68.5) |
| Female | 68(28.2) | 24(38.1) | 14(27.5) | 17(48.6) | 16(37.2) | 14(26.4) | 153(31.5) |
| *Age (years)* |  |  |  |  |  |  |  |
| 16-25 | 5(2.1) | 1(1.6) | 0(0.0) | 0(0.0) | 3(7.0) | 0(0.0) | 9(1.9) |
| 26-35 | 21(8.7) | 7(11.1) | 7(13.7) | 7(20.0) | 5(11.6) | 0(0.0) | 47(9.7) |
| 36-45 | 51(21.2) | 13(20.6) | 7(13.7) | 6(17.1) | 14(32.6) | 5(9.4) | 96(19.8) |
| 46-55 | 119(49.4) | 34(54.0) | 31(60.8) | 21(60.0) | 17(39.5) | 38(71.7) | 260(53.5) |
| 56 and above | 45(18.7) | 8(12.7) | 6(11.8) | 1(2.9) | 4(9.3) | 10(18.9) | 74(15.2) |

Table S4: Farmers who have received formal or informal training on pesticide use/application

|  | Uyo | IkotEkpene | Abak | Etinan | Eket | Oron | Total |
| --- | --- | --- | --- | --- | --- | --- | --- |
| Trained | 43(19.0) | 51(85.0) | 21(46.7) | 26(74.3) | 18(46.2) | 11(23.4) | 170(37.6) |
| Not trained | 183(81.0) | 9(15.0) | 24(53.3) | 9(25.7) | 21(53.8) | 36(76.6) | 286(62.4) |

Table S5: Percentage frequency of pesticides used in six agricultural zones of AkwaIbom State

| Commercial/local names | Pesticide class | Chemical content or mixture | Uyo | Ikot Ekpene | Abak | Etinan | Eket | Oron | Total |
| --- | --- | --- | --- | --- | --- | --- | --- | --- | --- |
| Decis | Pyrethroid | Detthamethrin | 36(14.9) | 6(9.5) | 8(15.7) | 16(45.7) | 10(23.3) | 0(0.0) | 76(15.6) |
| Attack | Organophosphate/  Pyrethroid | Chloropyrifos (50%) + cypermethrin (50%) | 11(4.6) | 3(4.8) | 4(7.8) | 2(5.7) | 1(2.3) | 0(0.0) | 21(4.3) |
| DD force | Organophosphate | Dichlorovos | 7(2.9) | 5(7.9) | 8(15.7) | 1(2.9) | 8(18.6) | 1(1.9) | 30(6.2) |
| Termex | Organochlorine | Imidacloprid | 70(29.0) | 3(4.8) | 9(17.6) | 5(14.3) | 1(2.3) | 46(86.8) | 134(6.2) |
| Kombat | Pyrethroid | Cyhalothrin | 0(0.0) | 0(0.0) | 0(0.0) | 0(0.0) | 1(2.3) | 0(0.0) | 1(0.2) |
| Furadan or carbofuran | Carbamate | Carbofuran | 3(1.2) | 1(1.6) | 3(5.9) | 3(8.6) | 1(2.3) | 4(7.5) | 15(3.1) |
| Karate | Pyrethroid | Cyhalothrin | 4(1.7) | 10(15.8) | 3(5.9) | 16(45.7) | 15(34.9) | 3(5.7) | 51(10.5) |
| Apron-plus | Acylalanine fungicide | Metalaxyl | 5(2.1) | 1(1.6) | 4(7.8) | 0(0.0) | 1(2.3) | 1(1.9) | 12(2.5) |
| Nuvacron | Organophosphate | Monocrotophos | 0(0.0) | 0(0.0) | 0(0.0) | 0(0.0) | 0(0.0) | 1(1.9) | 1(0.2) |
| Aldrin | Organochlorine | Chlorinated cyclodienes | 1(0.4) | 0(0.0) | 1(2.0) | 0(0.0) | 0(0.0) | 1(1.9) | 3(0.6) |
| Cyper Force | Pyrethroid | Pyrethroid | 0(0.0) | 7(11.1) | 0(0.0) | 0(0.0) | 2(4.7) | 1(1.9) | 10(2.1) |
| Rambo insect power | Pyrethroid | Permethrin | 2(0.8) | 0(0.0) | 0(0.0) | 0(0.0) | 1(2.3) | 2(3.8) | 5(1.0) |
| Dimetrin | Pyrethroid | Pyrethroid | 0(0.0) | 0(0.0) | 0(0.0) | 0(0.0) | 0(0.0) | 1(1.9) | 1(0.2) |
| Super Karto | Pyrethroid | Cyhalothrin | 0(0.0) | 14(22.2) | 0(0.0) | 1(2.9) | 0(0.0) | 1(1.9) | 16(3.3) |
| Ambush | Pyrethroid | Permethrin | 0(0.0) | 0(0.0) | 0(0.0) | 0(0.0) | 0(0.0) | 1(11.3) | 1(0.2) |
| Acuron 40 | Bicyclopyrone herbicide | Metachlor, mesotrione, bycyclopyrone, atrazine | 0(0.0) | 2(3.2) | 0(0.0) | 0(0.0) | 1(2.3) | 1(11.3) | 4(0.8) |
| Best | Unknown | Unknown | 14(5.8) | 3(4.8) | 1(2.0) | 2(5.7) | 2(4.6) | 46(86.8) | 68(14.0) |
| Busadin | Unknown | Unknown | 4(1.7) | 0(0.0) | 0(0.0) | 0(0.0) | 0(0.0) | 0(0.0) | 4(0.8) |
| Premex | Organochlorine | Chlorothalonil + oxathiapiprolin | 3(1.2) | 7(11.1) | 0(0.0) | 0(0.0) | 0(0.0) | 1(1.9) | 11(2.3) |
| Gramazon | Organochlorine | Paraquat | 4(1.7) | 0(0.0) | 0(0.0) | 0(0.0) | 2(4.6) | 48(90.6) | 54(11.1) |
| Others | - | - | 58(24.1) | 23(36.5) | 6(11.8) | 9(25.7) | 6(14.0) | 4(7.5) | 106(21.8) |

Table S6: Most important economic crops for pesticide application in Akwa Ibom State

|  | Uyo | Ikot Ekpene | Abak | Etinan | Eket | Oron | Total |
| --- | --- | --- | --- | --- | --- | --- | --- |
| Cassava | 85(35.4) | 33(38.1) | 23(45.1) | 12(34.3) | 25(58.1) | 52(98.1) | 230(47.4) |
| Maize | 166(69.2) | 39(61.9) | 20(39.2) | 16(45.7) | 19(44.2) | 49(92.5) | 309(63.7) |
| Pumpkin | 162(67.5) | 29(46.0) | 27(52.9) | 23(65.7) | 26(60.5) | 50(94.3) | 317(65.7) |
| Okra | 4(1.7) | 0(0.0) | 3(6.0) | 1(2.9) | 2(4.7) | 1(1.9) | 11(2.3) |
| Plantain | 0(0.0) | 0(0.0) | 2(4.9) | 0(0.0) | 1(2.3) | 2(3.8) | 5(1.0) |
| melon | 28(11.7) | 13(20.6) | 5(9.8) | 1(2.9) | 9(20.9) | 2(3.8) | 58(12.0) |
| Cucumber | 11(4.6) | 1(1.6) | 1(2.0) | 1(2.9) | 1(2.3) | 2(3.8) | 17(3.5) |
| Waterleaf | 0(0.0) | 0(0.0) | 0(0.0) | 1(2.9) | 1(2.3) | 1(1.9) | 3(0.6) |
| Tomatoes | 12(5.0) | 2(3.2) | 1(2.0) | 4(11.4) | 0(0.0) | 3(5.7) | 22(4.5) |
| Pepper | 4(1.7) | 1(1.6) | 0(0.0) | 3(8.6) | 2(4.7) | 1(1.9) | 11(2.3) |
| Others | 72(29.9) | 15(23.8) | 13(25.5) | 2(5.7) | 7(16.3) | 48(90.6) | 157(32.3) |

Table S7: Use of personal protective equipment during pesticide application in Akwa Ibom

|  | Uyo | Ikot Ekpene | Abak | Etinan | Eket | Oron | Total |
| --- | --- | --- | --- | --- | --- | --- | --- |
| Used | 102(50.7) | 51(92.7) | 27(84.4) | 30(88.2) | 29(78.4) | 29(87.9) | 268(68.4) |
| Not used | 99(49.3) | 4(7.3) | 5(15.6) | 4(11.8) | 8(21.6) | 4(12.1) | 124(31.6) |

Table S8: Method of pesticides application in Akwa Ibom State

|  | Uyo | Ikot Ekpene | Abak | Etinan | Eket | Oron | Total |
| --- | --- | --- | --- | --- | --- | --- | --- |
| Hand pump | 42(23.0) | 13(20.6) | 8(25.8) | 4(12.1) | 4(12.1) | 3(42.9) | 74(20.9) |
| Knapsack | 141(77.0) | 50(79.4) | 23(74.2) | 2.9(87.9) | 33(89.2) | 4(57.1) | 280(79.1) |

Table S9: Farmers’ opinion on period in which pesticides will remain active

|  | Uyo | IkotEkpene | Abak | Etinan | Eket | Oron | Total |
| --- | --- | --- | --- | --- | --- | --- | --- |
| Not known | 17(8.9) | 2(3.4) | 4(13.8) | 1(3.0) | 0(0.0) | 50(96.2) | 74(18.5) |
| On maturity / harvesting | 17(8.9) | 18(30.5) | 12(41.4) | 12(36.4) | 14(37.8) | 1(1.9) | 74(18.5) |
| One planting season | 104(54.5) | 34(57.6) | 11(37.9) | 25(45.5) | 22(59.5) | 1(1.9) | 187(46) |
| Others | 53(27.7) | 5(8.5) | 2(6.9) | 5(15.2) | 1(2.7) | 0(0.0) | 66(16.5) |

Table S10: Quantity (dosage) of pesticides use persquare metres of farmland

|  | Uyo | IkotEkpene | Abak | Etinan | Eket | Oron | Total |
| --- | --- | --- | --- | --- | --- | --- | --- |
| 20-25ml | 61(45.9) | 1(2.6) | 0(0.0) | 2(9.5) | 0(0.0) | 2(4.0) | 66(23.9) |
| 51- 100ml | 17(12312.80 | 1(2.6) | 1(5.3) | 0(0.0) | 0(0.0) | 0(0.0) | 19(6.9) |
| 101-150ml | 28(21.1) | 13(34.2) | 8(23.8) | 5(23.8) | 6(40.0) | 0(0.0) | 60(21.7) |
| 151-250 ml | 27(20.3) | 23(60.5) | 13(61.9) | 13(61.9) | 9(60.0) | 48(96.0) | 127(46.0) |
| Above 250 ml | 0(0.0) | 0(0.0) | 1(4.8) | 1(4.8) | 0(0.0) | 0(0.0) | 4(1.4) |

Table S11: Frequency of pesticides application within a planting season in AkwaIbom

|  | Uyo | IkotEkpene | Abak | Etinan | Eket | Oron | Total |
| --- | --- | --- | --- | --- | --- | --- | --- |
| Once | 32(16.6) | 5(8.6) | 3(9.7) | 6(17.6) | 4(1.4) | 2(28.6) | 52(14.5) |
| Twice | 82(42.5) | 27(46.6) | 11(35.5) | 19(55.9) | 7(20.0) | 0(0.0) | 146(40.6) |
| 3 time | 8(4.1) | 11(19.0) | 5(16.1) | 3(8.8) | 4(11.4) | 0(0.0) | 31(8.7) |
| As need rises | 43(22.3) | 11(19.0) | 6(19.4) | 0(0.0) | 11(31.4) | 5(71.4) | 76(21.2) |
| >3 times | 28(14.5) | 4(6.9) | 6(19.4) | 6(17.6) | 9(25.7) | 0(0.0) | 53(14.8) |

Table S12: Time allowed after pesticides application before sending products to the market

|  | Uyo | IkotEkpene | Abak | Etinan | Eket | Oron | Total |
| --- | --- | --- | --- | --- | --- | --- | --- |
| 1 week | 32(16.0) | 9(15.3) | 1(3.1) | 1(2.9) | 2(5.4) | 0(0.0) | 45(12.2) |
| 2-3 weeks | 110(55.0) | 38(64.4) | 23(71.9) | 28(80.0) | 29(78.4) | 5(71.4) | 233(63.0) |
| 4 weeks and above | 58(29.0) | 12(20.3) | 8(25.0) | 6(17.1) | 6(16.2) | 2(28.6) | 92(24.9) |

Table S13: Levels of total OCP (µg/kg) residues detected in different soils and sediments in Nigeria and some other countries

| OCPs/Location | HCH | ƩHCH | DDT | ƩDDT | Chlordane | HCB | Ʃ Heptachlor | Dieldrin | Ʃ Endosulfan | Endrin | Aldrin | Source |
| --- | --- | --- | --- | --- | --- | --- | --- | --- | --- | --- | --- | --- |
| Mertijeju, Ethiopia - Soil | NR | NR | 1.5-11 | NR | NR | NR | ND | <0.7-2.4 | <1.2-28900 | ND-6.3 | ND | [60] |
| Nuraera, Ethiopia - Soil | ND-5.5 | NR | <0.7-2.3 | NR | NR | NR | ND-<1.8 | <0.7 | <1.2-72 | ND-<6.3 | ND-<3.8 | [60] |
| Illushi, Edo, Nigeria | 1.20-2.17 | 4.08-4.90 | 0.93-1.16 | NR | NR | NR | NR | NR | NR | NR | NR | [61] |
| Midway Atoll, Hawai | ND-83.7 | ND-127 | ND-359 | 1.4-643 | NR | NR | NR | NR | NR | NR | NR | [62] |
| Azerbaijan | NR | 0.9-25 | NR | 1-1115 | NR | NR | NR | NR | NR | NR | NR | [63] |
| James Ross Island, Antarctica | NR | 0.5-13 | NR | 0.5-3.7 | NR | NR | NR | NR | NR | NR | NR | [64] |
| Cameron highland, Malaysia | NR | 0.00806 | NR | 0.0065 | NR | NR | NR | NR | NR | NR | NR | [65] |
| FADAMA farm, Minna, NG | NR | NR | 0.289-0.296 | | NR | NR | NR | NR | 0.049-0.056 | NR | NR | [66] |
| New Territories, Hong Kong | 1.14-26.8 | NR | 2.04-64.7 | NR | NR | NR | NR | NR | NR | NR | NR | [53] |
| Ningde, Southeast China | 0.45-151.21 | NR | 0.51-241.30 | NR | NR | NR | NR | NR | 0.14-453.96 | NR | NR | [17] |
| North of London, UK | ND-25 | NR | ND-85 | NR | NR | 0.14-7 | NR | <0.03-4.9 | NR | NR | NR | [67] |
| NW China | 0.17-9.38 | NR | 0.10-120.49 | NR | ND-0.28 | ND-11.17 | 0.02-0.21 | NR | 0.01-0.84 | NR | NR | [12] |
| Mbarali-mbeya, Tanzania | 0.04-11 | NR | 15-372 | NR | ND | 0.03-0.64 | ND | 0.02-1.04 | 0.01-4.7 | ND-0.001 | 0.0004-0.02 | [68] |

ND = Not Detected, NR = Not Reported; NG: Nigeria

Table S14: Pearson correlation matrix of OCPs

|  | **1** | **2** | **3** | **4** | **5** | **6** | **7** | **8** | **9** | **10** | **11** | **12** | **13** |
| --- | --- | --- | --- | --- | --- | --- | --- | --- | --- | --- | --- | --- | --- |
| **1.** α -HCH | 1 |  |  |  |  |  |  |  |  |  |  |  |  |
| **2.**β-HCH | -.197 | 1 |  |  |  |  |  |  |  |  |  |  |  |
| **3.**γ-HCH | -.098 | .250 | 1 |  |  |  |  |  |  |  |  |  |  |
| **4.**δ-HCH | -.134 | -.094 | .099 | 1 |  |  |  |  |  |  |  |  |  |
| **5.**HCB | -.207 | -.065 | .253 | .113 | 1 |  |  |  |  |  |  |  |  |
| **6.**o,p’ DDD | -.134 | .440^*^ | .263 | -.027 | -.066 | 1 |  |  |  |  |  |  |  |
| **7.**p,p’ DDD | -.266 | .588^**^ | .164 | -.085 | -.208 | .745^**^ | 1 |  |  |  |  |  |  |
| **8.**o,p’ DDE | -.082 | .221 | .219 | -.054 | -.019 | -.156 | -.149 | 1 |  |  |  |  |  |
| **9.**p,p’ DDE | .392 | .170 | .275 | -.100 | -.051 | .306 | .346 | -.252 | 1 |  |  |  |  |
| **10.**o,p’ DDT | -.068 | .348 | .187 | -.071 | -.140 | .551^**^ | .648^**^ | -.111 | .093 | 1 |  |  |  |
| **11.**p,p’ DDT | -.055 | .115 | -.009 | -.047 | -.141 | .267 | .534^**^ | -.171 | .361 | .409^*^ | 1 |  |  |
| **12.**α -Chlordane | -.136 | .100 | .070 | -.088 | -.046 | .259 | .384 | -.122 | .357 | .433^*^ | .502^*^ | 1 |  |
| **13.**γ-Chlordane | -.114 | .286 | .225 | -.137 | .028 | -.099 | -.061 | .977^**^ | -.135 | -.089 | -.106 | -.037 | 1 |

| *. Correlation is significant at the 0.05 level (2-tailed). |
| --- |
| **. Correlation is significant at the 0.01 level (2-tailed). |

Table S15: Average daily dose (ADDingest) (mg/kg/day) for adult

| OCPs | Abak | Ikot Ekpene | Etinan | Eket | Oron | Uyo | Control |
| --- | --- | --- | --- | --- | --- | --- | --- |
| ƩHCH | 3.34E-09 | 4.04E-09 | 1.44E-08 | 6.11E-09 | 2.86E-08 | 1.44E-08 | 4.52E-09 |
| ƩDDD | 1.99E-09 | 4.79E-10 | 1.62E-09 | 5.07E-10 | 9.59E-11 | 9.45E-10 | 5.34E-10 |
| ƩDDE | 3.84E-10 | 2.60E-10 | 3.29E-10 | 2.47E-10 | 5.34E-10 | 1.51E-10 | 8.22E-11 |
| ƩDDT | 5.63E-09 | 5.89E-10 | 8.08E-10 | 9.59E-10 | 4.66E-10 | 1.75E-09 | 1.64E-10 |
| ƩChlordane | 6.85E-11 | 1.37E-11 | 2.74E-11 | 5.48E-11 | 0.00E+00 | 0.00E+00 | 0.00E+00 |
| HCB | 2.74E-11 | 4.52E-10 | 1.23E-10 | 5.48E-11 | 1.37E-11 | 1.37E-11 | 1.37E-11 |

Table S16: Average daily dose (ADDingest) (mg/kg/day) for children

| OCPs | Abak | Ikot Ekpene | | Etinan | | Eket | | Oron | | Uyo | | Control | |
| --- | --- | --- | --- | --- | --- | --- | --- | --- | --- | --- | --- | --- | --- |
| ƩHCH | 3.25E-09 | 3.93E-09 | 1.40E-08 | | 5.94E-09 | | 2.78E-08 | | 1.40E-08 | | 4.39E-09 | |  |
| ƩDDD | 1.93E-09 | 4.66E-10 | 1.57E-09 | | 4.93E-10 | | 9.32E-11 | | 9.19E-10 | | 5.19E-10 | |  |
| ƩDDE | 3.73E-10 | 2.53E-10 | 3.20E-10 | | 2.40E-10 | | 5.19E-10 | | 1.46E-10 | | 7.99E-11 | |  |
| ƩDDT | 5.47E-09 | 5.73E-10 | 7.86E-10 | | 9.32E-10 | | 4.53E-10 | | 1.70E-09 | | 1.60E-10 | |  |
| ƩChlordane | 6.66E-11 | 1.33E-11 | 2.66E-11 | | 5.33E-11 | | 0.00E+00 | | 0.00E+00 | | 0.00E+00 | |  |
| HCB | 2.66E-11 | 4.39E-10 | 1.20E-10 | | 5.33E-11 | | 1.33E-11 | | 1.33E-11 | | 1.33E-11 | |  |

Table S17: Average daily dose (ADDdermal) (mg/kg/day) for adulthood

| OCPs | Abak | Ikot Ekpene | Etinan | Eket | Oron | Uyo | Control |
| --- | --- | --- | --- | --- | --- | --- | --- |
| ƩHCH | 1.73E-09 | 2.10E-09 | 7.45E-09 | 3.17E-09 | 1.48E-08 | 7.47E-09 | 2.34E-09 |
| ƩDDD | 1.03E-09 | 2.49E-10 | 8.38E-10 | 2.63E-10 | 4.97E-11 | 4.90E-10 | 2.77E-10 |
| ƩDDE | 1.99E-10 | 1.35E-10 | 1.71E-10 | 1.28E-10 | 2.77E-10 | 7.82E-11 | 4.26E-11 |
| ƩDDT | 2.92E-09 | 3.06E-10 | 4.19E-10 | 4.97E-10 | 2.42E-10 | 9.10E-10 | 8.53E-11 |
| ƩChlordane | 3.55E-11 | 7.11E-12 | 1.42E-11 | 2.84E-11 | 0.00E+00 | 0.00E+00 | 0.00E+00 |
| HCB | 1.42E-11 | 2.34E-10 | 6.39E-11 | 2.84E-11 | 7.11E-12 | 7.11E-12 | 7.11E-12 |

Table S18: Average daily dose (ADDdermal) (mg/kg/day) for childhood

| OCPs | Abak | Ikot Ekpene | Etinan | Eket | Oron | Uyo | Control |
| --- | --- | --- | --- | --- | --- | --- | --- |
| ƩHCH | 1.18E-09 | 1.43E-09 | 5.09E-09 | 2.16E-09 | 1.01E-08 | 5.10E-09 | 1.60E-09 |
| ƩDDD | 7.03E-10 | 1.70E-10 | 5.72E-10 | 1.79E-10 | 3.39E-11 | 3.34E-10 | 1.89E-10 |
| ƩDDE | 1.36E-10 | 9.21E-11 | 1.16E-10 | 8.73E-11 | 1.89E-10 | 5.33E-11 | 2.91E-11 |
| ƩDDT | 1.99E-09 | 2.08E-10 | 2.86E-10 | 3.39E-10 | 1.65E-10 | 6.21E-10 | 5.82E-11 |
| ƩChlordane | 2.42E-11 | 4.85E-12 | 9.70E-12 | 1.94E-11 | 0.00E+00 | 0.00E+00 | 0.00E+00 |
| HCB | 9.70E-12 | 1.60E-10 | 4.36E-11 | 1.94E-11 | 4.85E-12 | 4.85E-12 | 4.85E-12 |

Table S19: Average daily dose (ADDinhale) (mg/kg/day) for adulthood

| OCPs | Abak | Ikot Ekpene | Etinan | Eket | Oron | Uyo | Control |
| --- | --- | --- | --- | --- | --- | --- | --- |
| ƩHCH | 2.80E-13 | 3.39E-13 | 1.20E-12 | 5.12E-13 | 2.39E-12 | 1.21E-12 | 3.79E-13 |
| ƩDDD | 1.67E-13 | 4.02E-14 | 1.36E-13 | 4.25E-14 | 8.04E-15 | 7.93E-14 | 4.48E-14 |
| ƩDDE | 3.22E-14 | 2.18E-14 | 2.76E-14 | 2.07E-14 | 4.48E-14 | 1.26E-14 | 6.89E-15 |
| ƩDDT | 4.72E-13 | 4.94E-14 | 6.78E-14 | 8.04E-14 | 3.91E-14 | 1.47E-13 | 1.38E-14 |
| ƩChlordane | 5.74E-15 | 1.15E-15 | 2.30E-15 | 4.59E-15 | 0.00E+00 | 0.00E+00 | 0.00E+00 |
| HCB | 2.30E-15 | 3.79E-14 | 1.03E-14 | 4.59E-15 | 1.15E-15 | 1.15E-15 | 1.15E-15 |

Table S20: Average daily dose (ADDinhale) (mg/kg/day) for childhood

| OCPs | Abak | Ikot Ekpene | Etinan | Eket | Oron | Uyo | Control |
| --- | --- | --- | --- | --- | --- | --- | --- |
| ƩHCH | 1.36E-13 | 1.65E-13 | 5.86E-13 | 2.49E-13 | 1.16E-12 | 5.87E-13 | 1.84E-13 |
| ƩDDD | 8.10E-14 | 1.95E-14 | 6.59E-14 | 2.07E-14 | 3.91E-15 | 3.85E-14 | 2.18E-14 |
| ƩDDE | 1.56E-14 | 1.06E-14 | 1.34E-14 | 1.01E-14 | 2.18E-14 | 6.14E-15 | 3.35E-15 |
| ƩDDT | 2.29E-13 | 2.40E-14 | 3.29E-14 | 3.91E-14 | 1.90E-14 | 7.15E-14 | 6.70E-15 |
| ƩChlordane | 2.79E-15 | 5.58E-16 | 1.12E-15 | 2.23E-15 | 0.00E+00 | 0.00E+00 | 0.00E+00 |
| HCB | 1.12E-15 | 1.84E-14 | 5.03E-15 | 2.23E-15 | 5.58E-16 | 5.58E-16 | 5.58E-16 |

Table S21: Hazard Quotient_ingest_ for childhood

| OCPs | Abak | Ikot Ekpene | Etinan | Eket | Oron | Uyo | Control | HI of the zone |
| --- | --- | --- | --- | --- | --- | --- | --- | --- |
| ƩHCH | 1.67E-13 | 2.02E-13 | 7.18E-13 | 3.05E-13 | 1.43E-12 | 7.21E-13 | 2.26E-13 | 3.77E-12 |
| ƩDDD | 3.97E-14 | 9.59E-15 | 3.23E-14 | 1.01E-14 | 1.92E-15 | 1.89E-14 | 1.07E-14 | 1.23E-13 |
| ƩDDE | 7.67E-15 | 5.21E-15 | 6.58E-15 | 4.93E-15 | 1.07E-14 | 3.01E-15 | 1.64E-15 | 3.97E-14 |
| ƩDDT | 1.13E-13 | 1.18E-14 | 1.62E-14 | 1.92E-14 | 9.32E-15 | 3.51E-14 | 3.29E-15 | 2.07E-13 |
| ƩChlordane | 1.37E-15 | 2.74E-16 | 5.48E-16 | 1.10E-15 | 0.00E+00 | 0.00E+00 | 0.00E+00 | 3.29E-15 |
| HCB | 3.42E-16 | 5.65E-15 | 1.54E-15 | 6.85E-16 | 1.71E-16 | 1.71E-16 | 1.71E-16 | 8.73E-15 |
| HI per location | 3.29E-13 | 2.35E-13 | 7.76E-13 | 3.42E-13 | 1.45E-12 | 7.78E-13 | 2.42E-13 |  |

Table S22: Hazard Quotientingest for adult

| OCPs | Abak | Ikot Ekpene | Etinan | Eket | Oron | Uyo | Control | **HI of the zone** |
| --- | --- | --- | --- | --- | --- | --- | --- | --- |
| ƩHCH | 1.62E-13 | 1.96E-13 | 6.99E-13 | 2.97E-13 | 1.39E-12 | 7.01E-13 | 2.20E-13 | **3.66E-12** |
| ƩDDD | 3.86E-14 | 9.32E-15 | 3.14E-14 | 9.86E-15 | 1.86E-15 | 1.84E-14 | 1.04E-14 | **1.20E-13** |
| ƩDDE | 7.46E-15 | 5.06E-15 | 6.39E-15 | 4.79E-15 | 1.04E-14 | 2.93E-15 | 1.60E-15 | **3.86E-14** |
| ƩDDT | 1.09E-13 | 1.15E-14 | 1.57E-14 | 1.86E-14 | 9.06E-15 | 3.41E-14 | 3.20E-15 | **2.02E-13** |
| ƩChlordane | 1.33E-15 | 2.66E-16 | 5.33E-16 | 1.07E-15 | 0.00E+00 | 0.00E+00 | 0.00E+00 | **3.20E-15** |
| HCB | 3.33E-16 | 5.49E-15 | 1.50E-15 | 6.66E-16 | 1.66E-16 | 1.66E-16 | 1.66E-16 | **8.49E-15** |
| HI per location | 3.20E-13 | 2.28E-13 | 7.54E-13 | 3.32E-13 | 1.41E-12 | 7.56E-13 | 2.35E-13 |  |

Table S23: Hazard Quotient (dermal) for children

| OCPs | Abak | Ikot Ekpene | Etinan | Eket | Oron | Uyo | Control | HI of the zone |
| --- | --- | --- | --- | --- | --- | --- | --- | --- |
| ƩHCH | 5.91E-14 | 7.15E-14 | 2.54E-13 | 1.08E-13 | 5.05E-13 | 2.55E-13 | 8.00E-14 | 1.33E-12 |
| ƩDDD | 1.41E-14 | 3.39E-15 | 1.14E-14 | 3.59E-15 | 6.79E-16 | 6.69E-15 | 3.78E-15 | 4.36E-14 |
| ƩDDE | 2.71E-15 | 1.84E-15 | 2.33E-15 | 1.75E-15 | 3.78E-15 | 1.07E-15 | 5.82E-16 | 1.41E-14 |
| ƩDDT | 3.98E-14 | 4.17E-15 | 5.72E-15 | 6.79E-15 | 3.30E-15 | 1.24E-14 | 1.16E-15 | 7.34E-14 |
| ƩChlordane | 4.85E-16 | 9.70E-17 | 1.94E-16 | 3.88E-16 | 0.00E+00 | 0.00E+00 | 0.00E+00 | 1.16E-15 |
| HCB | 1.21E-16 | 2.00E-15 | 5.45E-16 | 2.42E-16 | 6.06E-17 | 6.06E-17 | 6.06E-17 | 3.09E-15 |
| HI per location | 1.16E-13 | 8.30E-14 | 2.74E-13 | 1.21E-13 | 5.13E-13 | 2.75E-13 | 8.56E-14 |  |

Table S24: Hazard Quotient (dermal) for adult

| OCPs | Abak | Ikot Ekpene | Etinan | Eket | Oron | Uyo | Control | HI of the zone |
| --- | --- | --- | --- | --- | --- | --- | --- | --- |
| ƩHCH | 8.67E-14 | 1.05E-13 | 3.73E-13 | 1.58E-13 | 7.41E-13 | 3.74E-13 | 1.17E-13 | 1.95E-12 |
| ƩDDD | 2.06E-14 | 4.97E-15 | 1.68E-14 | 5.26E-15 | 9.95E-16 | 9.81E-15 | 5.54E-15 | 6.39E-14 |
| ƩDDE | 3.98E-15 | 2.70E-15 | 3.41E-15 | 2.56E-15 | 5.54E-15 | 1.56E-15 | 8.53E-16 | 2.06E-14 |
| ƩDDT | 5.84E-14 | 6.11E-15 | 8.38E-15 | 9.95E-15 | 4.83E-15 | 1.82E-14 | 1.71E-15 | 1.08E-13 |
| ƩChlordane | 7.11E-16 | 1.42E-16 | 2.84E-16 | 5.68E-16 | 0.00E+00 | 0.00E+00 | 0.00E+00 | 1.71E-15 |
| HCB | 1.78E-16 | 2.93E-15 | 7.99E-16 | 3.55E-16 | 8.88E-17 | 8.88E-17 | 8.88E-17 | 4.53E-15 |
| HI per location | 1.71E-13 | 1.22E-13 | 4.02E-13 | 1.77E-13 | 7.52E-13 | 4.03E-13 | 1.25E-13 |  |

Table S25: Hazard Quotient (inhale) for children

| OCPs | Abak | Ikot Ekpene | Etinan | Eket | Oron | Uyo | Control | HI of the zone |
| --- | --- | --- | --- | --- | --- | --- | --- | --- |
| ƩHCH | 6.81E-18 | 8.24E-18 | 2.93E-17 | 1.25E-17 | 5.82E-17 | 2.94E-17 | 9.21E-18 | 1.54E-16 |
| ƩDDD | 1.62E-18 | 3.91E-19 | 1.32E-18 | 4.13E-19 | 7.82E-20 | 7.71E-19 | 4.36E-19 | 5.03E-18 |
| ƩDDE | 3.13E-19 | 2.12E-19 | 2.68E-19 | 2.01E-19 | 4.36E-19 | 1.23E-19 | 6.70E-20 | 1.62E-18 |
| ƩDDT | 4.59E-18 | 4.80E-19 | 6.59E-19 | 7.82E-19 | 3.80E-19 | 1.43E-18 | 1.34E-19 | 8.45E-18 |
| ƩChlordane | 5.58E-20 | 1.12E-20 | 2.23E-20 | 4.47E-20 | 0.00E+00 | 0.00E+00 | 0.00E+00 | 1.34E-19 |
| HCB | 1.40E-20 | 2.30E-19 | 6.28E-20 | 2.79E-20 | 6.98E-21 | 6.98E-21 | 6.98E-21 | 3.56E-19 |
| HI per location | 1.34E-17 | 9.56E-18 | 3.16E-17 | 1.39E-17 | 5.91E-17 | 3.17E-17 | 9.86E-18 |  |

Table S26: Hazard Quotient (inhale) for adult

| OCPs | Abak | Ikot Ekpene | Etinan | Eket | Oron | Uyo | Control | HI of the zone |
| --- | --- | --- | --- | --- | --- | --- | --- | --- |
| ƩHCH | 1.40E-17 | 1.69E-17 | 6.02E-17 | 2.56E-17 | 1.20E-16 | 6.04E-17 | 1.90E-17 | 3.16E-16 |
| ƩDDD | 3.33E-18 | 8.04E-19 | 2.71E-18 | 8.50E-19 | 1.61E-19 | 1.59E-18 | 8.96E-19 | 1.03E-17 |
| ƩDDE | 6.43E-19 | 4.36E-19 | 5.51E-19 | 4.13E-19 | 8.96E-19 | 2.53E-19 | 1.38E-19 | 3.33E-18 |
| ƩDDT | 9.44E-18 | 9.88E-19 | 1.36E-18 | 1.61E-18 | 7.81E-19 | 2.94E-18 | 2.76E-19 | 1.74E-17 |
| ƩChlordane | 1.15E-19 | 2.30E-20 | 4.59E-20 | 9.19E-20 | 0.00E+00 | 0.00E+00 | 0.00E+00 | 2.76E-19 |
| HCB | 2.87E-20 | 4.74E-19 | 1.29E-19 | 5.74E-20 | 1.44E-20 | 1.44E-20 | 1.44E-20 | 7.32E-19 |
| HI per location | 2.76E-17 | 1.97E-17 | 6.50E-17 | 2.86E-17 | 1.22E-16 | 6.52E-17 | 2.03E-17 |  |

Table S27: Cancer Risk estimation due to ingestion for children

| OCPs | Abak | Ikot Ekpene | Etinan | Eket | Oron | Uyo | Control |
| --- | --- | --- | --- | --- | --- | --- | --- |
| ƩHCH | 1.81E-05 | 2.18E-05 | 7.76E-05 | 3.30E-05 | 1.54E-04 | 7.78E-05 | 2.44E-05 |
| ƩDDD | 4.09E-07 | 9.87E-08 | 3.33E-07 | 1.04E-07 | 1.97E-08 | 1.94E-07 | 1.10E-07 |
| ƩDDE | 1.12E-07 | 7.59E-08 | 9.58E-08 | 7.19E-08 | 1.56E-07 | 4.39E-08 | 2.40E-08 |
| ƩDDT | 1.64E-06 | 1.72E-07 | 2.36E-07 | 2.80E-07 | 1.36E-07 | 5.11E-07 | 4.79E-08 |
| ƩChlordane | 2.06E-08 | 4.11E-09 | 8.22E-09 | 1.64E-08 | 0.00E+00 | 0.00E+00 | 0.00E+00 |
| HCB | 3.75E-09 | 6.19E-08 | 1.69E-08 | 7.51E-09 | 1.88E-09 | 1.88E-09 | 1.88E-09 |

Table S28: Cancer Risk estimation due to ingestion for adult

| OCPs | Abak | Ikot Ekpene | Etinan | Eket | Oron | Uyo | Control |
| --- | --- | --- | --- | --- | --- | --- | --- |
| ƩHCH | 2.11E-05 | 2.55E-05 | 9.05E-05 | 3.85E-05 | 1.80E-04 | 9.08E-05 | 2.85E-05 |
| ƩDDD | 4.77E-07 | 1.15E-07 | 3.88E-07 | 1.22E-07 | 2.30E-08 | 2.27E-07 | 1.28E-07 |
| ƩDDE | 1.30E-07 | 8.85E-08 | 1.12E-07 | 8.38E-08 | 1.82E-07 | 5.12E-08 | 2.79E-08 |
| ƩDDT | 1.91E-06 | 2.00E-07 | 2.75E-07 | 3.26E-07 | 1.58E-07 | 5.96E-07 | 5.59E-08 |
| ƩChlordane | 2.40E-08 | 4.79E-09 | 9.59E-09 | 1.92E-08 | 0.00E+00 | 0.00E+00 | 0.00E+00 |
| HCB | 1.10E-07 | 2.19E-08 | 4.38E-08 | 8.77E-08 | 0.00E+00 | 0.00E+00 | 0.00E+00 |

Table S29: Cancer Risk estimation due to dermal exposure for children

| OCPs | Abak | Ikot Ekpene | Etinan | Eket | Oron | Uyo | Control |
| --- | --- | --- | --- | --- | --- | --- | --- |
| ƩHCH | 2.04E-06 | 2.46E-06 | 8.75E-06 | 3.72E-06 | 1.74E-05 | 8.78E-06 | 2.75E-06 |
| ƩDDD | 9.24E-08 | 2.23E-08 | 7.52E-08 | 2.36E-08 | 4.46E-09 | 4.40E-08 | 2.49E-08 |
| ƩDDE | 2.53E-08 | 1.72E-08 | 2.17E-08 | 1.63E-08 | 3.52E-08 | 9.93E-09 | 5.42E-09 |
| ƩDDT | 3.71E-07 | 3.88E-08 | 5.33E-08 | 6.32E-08 | 3.07E-08 | 1.16E-07 | 1.08E-08 |
| ƩChlordane | 6.50E-09 | 1.30E-09 | 2.60E-09 | 5.20E-09 | 0.00E+00 | 0.00E+00 | 0.00E+00 |
| HCB | 1.19E-08 | 1.96E-07 | 5.35E-08 | 2.38E-08 | 5.95E-09 | 5.95E-09 | 5.95E-09 |

Table S30: Cancer Risk estimation due to dermal exposure for adults

| OCPs | Abak | Ikot Ekpene | Etinan | Eket | Oron | Uyo | Control |
| --- | --- | --- | --- | --- | --- | --- | --- |
| ƩHCH | 4.75E-06 | 5.74E-06 | 2.04E-05 | 8.68E-06 | 4.06E-05 | 2.05E-05 | 6.42E-06 |
| ƩDDD | 2.16E-07 | 5.20E-08 | 1.75E-07 | 5.50E-08 | 1.04E-08 | 1.03E-07 | 5.80E-08 |
| ƩDDE | 5.90E-08 | 4.00E-08 | 5.06E-08 | 3.79E-08 | 8.22E-08 | 2.32E-08 | 1.26E-08 |
| ƩDDT | 8.66E-07 | 9.06E-08 | 1.24E-07 | 1.47E-07 | 7.16E-08 | 2.70E-07 | 2.53E-08 |
| ƩChlordane | 1.52E-08 | 3.03E-09 | 6.07E-09 | 1.21E-08 | 0.00E+00 | 0.00E+00 | 0.00E+00 |
| HCB | 2.77E-08 | 4.58E-07 | 1.25E-07 | 5.55E-08 | 1.39E-08 | 1.39E-08 | 1.39E-08 |

Table S31: Cancer Risk estimation due to inhalation for children

| OCPs | Abak | Ikot Ekpene | Etinan | Eket | Oron | Uyo | Control |
| --- | --- | --- | --- | --- | --- | --- | --- |
| ƩHCH | 7.23E-07 | 8.75E-07 | 3.11E-06 | 1.32E-06 | 6.18E-06 | 3.12E-06 | 9.78E-07 |
| ƩDDD | NA | NA | NA | NA | NA | NA | NA |
| ƩDDE | NA | NA | NA | NA | NA | NA | NA |
| ƩDDT | 6.58E-08 | 6.88E-09 | 9.44E-09 | 1.12E-08 | 5.44E-09 | 2.05E-08 | 1.92E-09 |
| ƩChlordane | 8.24E-10 | 1.65E-10 | 3.29E-10 | 6.59E-10 | 0.00E+00 | 0.00E+00 | 0.00E+00 |
| HCB | NA | NA | NA | NA | NA | NA | NA |

Table S32: Cancer Risk estimation due to inhalation for adult

| OCPs | Abak | Ikot Ekpene | Etinan | Eket | Oron | Uyo | Control |
| --- | --- | --- | --- | --- | --- | --- | --- |
| ƩHCH | 1.69E-06 | 2.04E-06 | 7.26E-06 | 3.08E-06 | 1.44E-05 | 7.28E-06 | 2.28E-06 |
| ƩDDD | NA | NA | NA | NA | NA | NA | NA |
| ƩDDE | NA | NA | NA | NA | NA | NA | NA |
| ƩDDT | 1.53E-07 | 6.88E-09 | 9.44E-09 | 1.12E-08 | 5.44E-09 | 2.05E-08 | 1.92E-09 |
| ƩChlordane | 1.92E-09 | 3.84E-10 | 7.69E-10 | 1.54E-09 | 0.00E+00 | 0.00E+00 | 0.00E+00 |
| HCB | NA | NA | NA | NA | NA | NA | NA |

**References**

17. Qu, C., Qi, S., Yang, D., Huang, H., Zhang, J., Chena, W., Yohannes, H. K., Sandy, E. H., Yang, J., and Xing, X., 2015. Risk assessment and influence factors of organochlorine pesticides (OCPs) in agricultural soils of the hill region: A case study from Ningde, southeast China. *Journal of Geochemical Exploration*, 149: 43–51. <https://doi.org/10.1016/j.gexplo.2014.11.002>

52. Chen, S.C. and Liao, C.M., 2006. Health risk assessment on human exposed to environmental polycyclic aromatic hydrocarbons pollution sources. Science of the total environment, 366(1), pp.112-123. <https://doi.org/10.1016/j.scitotenv.2005.08.047>

53. Man, Y. B., Chow, K. L., Wang, H. S., Lau, K. Y., Sun, X. L., Wu, S. C., Cheung, K. C., Chung, S. S. and Wong, M. H., 2011. Health risk assessment of organochlorine pesticides with emphasis on DDTs and HCHs in abandoned agricultural soils. *Journal of Environmental Monitoring,* 13(8), 22-50. <https://doi.org/10.1039/C1EM10168D>

60. Westbom, R., Hussen, A., Megersa, N., Retta, N., Mathiasson, L. and Björklund, E. (2008). Assessment of organochlorine pesticide pollution in Upper Awash Ethiopian state farm soils using selective pressurised liquid extraction. *Chemosphere*, 72(8): 1181–1187. <https://doi.org/10.1016/j.chemosphere.2008.03.041>

61. Ogbeide, O., Tongo, I., Enuneku, A., Ogbomida, E., and Ezemonye, L., 2016b. Human health risk associated with dietary and non-dietary intake of organochlorine pesticide residues from rice fields in Edo State, Nigeria. *Exposure and Health*, 8(1): 53–66. <https://doi.org/10.1007/s12403-015-0182-6>

62. Ge, J., Woodward, L. A., Li, Q. X. and Wang, J., 2013. Composition, distribution and risk assessment of organochlorine pesticides in soils from the Midway Atoll, North Pacific Ocean. *Science of the Total Environment*, 452–453: 421–426. <https://doi.org/10.1016/j.scitotenv.2013.03.015>

63. Aliyeva, G., Kurkova, R., Hovorkova, I., Klanova, J. and Halsall, C., 2012. Organochlorine pesticides and polychlorinated biphenyls in air and soil across Azerbaijan*. Environmental Science and Pollution Research,* 19: 1956-1962. <https://doi.org/10.1007/s11356-012-0944-7>

64. Klanova, J., Matykiewiczová, N., Mácka, Z., Prosek, P., Láska, K. and Klán, P., 2008. Persistent organic pollutants in soils and sediments from James Ross Island, Antarctica. *Environmental Pollution*, 152(2): 416-23. <https://doi.org/10.1016/j.envpol.2007.06.026>

65. Saadati, N., Abdullah, M. P., Zakaria, Z., Rezayi, M., & Hosseinizare, N., 2012. Distribution and fate of HCH isomers and DDT metabolites in a tropical environment–case study Cameron Highlands–Malaysia. *Chemistry Central Journal*, 6, 1-15. <https://doi.org/10.1186/1752-153X-6-130>

66. Chikezie, O. I., Abdullahi, M., Jonathan, Y., and Abdulalahi, B., 2017. Assessment of organochlorine pesticide residues in soil and water from FADAMA Farming Communities in Minna, North Central, Nigeria. *AASCIT Journal of Environment*, 2(5): 48–55.

67. Meijer, S. N., Harner, T., Helm, P. A., Halsall, C. J., Johnston, A. E., & Jones, K. C., 2001. Polychlorinated naphthalenes in UK soils: time trends, markers of source, and equilibrium status. *Environmental science & technology*, 35(21), 4205-4213. <https://doi.org/10.1021/es010071d>

12. Huang, T., Guo, Q., Tian, H., Mao, X., Ding, Z., Zhang, G., Li, J., Ma, J. and Gao, H., 2014. Assessing spatial distribution, sources, and human health risk of organochlorine pesticide residues in the soils of arid and semiarid areas of Northwest China. *Environmental Science and Pollution Research,* 21(9), 6124–6135. <https://doi.org/10.1007/s11356-014-2505-8>

68. Mahugija, J. A. M., Henkelmann, B. and Schramm, K. W. (2014). Levels, compositions and distributions of organochlorine pesticide residues in soil 5-14 years after clean-up of former storage sites in Tanzania. *Chemosphere*, 117(1): 330–337. <https://doi.org/10.1016/j.chemosphere.2014.07.052>
